# Development, Cross-Cultural Adaptation, and Psychometric Characteristics of the Persian Progressive Aphasia Language Scale in Patients With Primary Progressive Aphasia: A Pilot Study

**DOI:** 10.29252/NIRP.BCN.9.1.35

**Published:** 2018

**Authors:** Salime Jafari, Amin Modarresszadeh, Ahmad Reza Khatoonabadi, John Hodges, Noureddin Nakhostin Ansari, Cristian Leyton, Maryam Noroozian

**Affiliations:** 1. Department of Speech Therapy, School of Rehabilitation, Tehran University of Medical Sciences, Tehran, Iran.; 2. Brain & Mind Centre, The University of Sydney, Sydney, Australia.; 3. Department of Physiotherapy, School of Rehabilitation, Tehran University of Medical Sciences, Tehran, Iran.; 4. Neuro-Musculoskeletal Research Center, Iran University of Medical Sciences, Tehran, Iran.; 5. Faculty of Health Sciences, The University of Sydney, Sydney, Australia.; 6. Memory and Behavioral Neurology Division, Roozbeh Hospital, Tehran University of Medical Sciences, Tehran, Iran.

**Keywords:** Aphasia, Neurodegenerative disorders, Primary progressive aphasia, Language test

## Abstract

**Introduction:**

Primary Progressive Aphasia (PPA) is a neurological condition characterized by progressive dissolution of language capabilities. The Progressive Aphasia Language Scale (PALS) is an easy-to-apply bedside clinical scale capable of capturing and grading the key language features essential for the classification of PPA. The objective of the present study was to develop and validate the Persian version of the PALS (PALS-P) as a clinical language assessment test.

**Methods:**

In this cross-sectional study, PALS was translated and adapted into Persian according to the international guidelines. A total of 30 subjects (10 subjects with PPA and 20 control subjects without dementia) were recruited to evaluate the intra-rater reliability and discriminant validity of PALS-P.

**Results:**

The intra-rater reliability of the PALS-P within a 14-day interval was excellent for each subtest (ICC agreement range=0.81–1.0). PALS-P results were statistically significant among groups, suggesting its discriminative validity.

**Conclusion:**

This preliminary study indicates that PALS-P was successfully developed and translated. It seems to be a valid and reliable screening tool to assess language skills in Persian-speaking subjects with progressive aphasia.

## 1. Introduction

Primary Progressive Aphasia (PPA) refers to a group of neurodegenerative disorders that slowly and progressively impair language function while sparing other aspects of cognitive processing such as memory, attention, visuospatial skills, and executive functions in the initial stages of the condition (Mesulam, 1982). According to a recently published framework for the diagnosis and classification of PPA, three clinical variants of PPA have been described: semantic dementia, progressive nonfluent aphasia, and progressive logopenic aphasia (Gorno Tempini et al., 2011).

Although a vast majority of cases fit in this tripartite classification, some cases with mild or mixed deficits make this classification clinically challenging. Therefore, researchers suggest an unclassified or mixed PPA variant as a fourth category for those patients with overlapping deficits (Leyton & Hodges, 2014; Mesulam et al., 2012). These three variants differently affect cortical regions responsible for the language network functions, which subsequently results in various clinical profiles (Gorno-Tempini et al., 2011; Mesulam et al., 2017). Each variant of primary progressive aphasia is associated with a different anatomical site of peak atrophy in the left-hemisphere language network: the inferior frontal gyrus (Boca’s area) in the agrammatic variant, the temporoparietal junction (Wernicke’s area) in the logopenic variant, and the anterior temporal lobe in the semantic variant. In semantic dementia, preferential atrophy of the anterior temporal lobe also occurs but usually in a more symmetric pattern that involves both hemispheres (Mesulam et al., 2017). Therefore, the cardinal feature of all PPA types is the deterioration of language functions while preserving other cognitive functions; the diagnosis of its variants must be based on the presence or absence of key speech/language features. Table 1 presents those language features considered by the International Consensus Group on PPA to be of cardinal importance for diagnosing the disorder and defining each of its variants.

**Table 1.** Summary of the core language features of PPA and its variants recommended by Gorno-Tempin et al. (2011) (adapted from Leyton et al., 2011)

**Core Language Features**	**Variants**
Primary progressive aphasia	1. Deficient language ability (i.e. word-finding deficits, effortful speech, paraphasias, grammatical and/or comprehension deficits) is the most prominent clinical feature of the disorder.
	2. Aphasia must be the most prominent impairment at the onset and remain the most significant deficit during the initial stages of the disorder.
	3. Present language deficits should not better accounted for by other conditions such as non-degenerative or psychiatric disorders.
Semantic variant	1. Dissolution of semantic knowledge is evident in poor confrontation naming and impaired single-word confrontation. 2. There is no sign of motor speech disorders or agrammatism.
Non-fluent variant	1. Either agrammatism or motor speech disorders in the form of effortful, halting speech, inconsistent sound errors and distortions is evident. 2. Single-word comprehension and object knowledge are preserved.
Logopenic variant	1. The flow of spontaneous speech and confrontational naming are interrupted by impaired single-word retrieval (word-finding pauses). Poor repetition of sentences and phrases is also evident. 2. Single-word comprehension is spared and there are no signs of motor speech disorders.

Despite the importance of considering speech and language criteria in the diagnosis of PPA and its variants, the assessment process may not be so straightforward in real clinical situations due to the potential inconsistency of signs and diagnostic biases. This is especially the case when a clinician is not well versed in the assessment of affected patients (Leyton et al., 2011). The problem may be partially resolved by using standardized tests that are able to quantify speech and language deficits (e.g. Western Aphasia Battery [WAB], Boston Diagnostic Aphasia Examination [BDEA]) (Goodglass, Kaplan & Barresi, 2000; Kertesz, 1982). WAB and BDEA are based on speech/language profiles of aphasic patients affected by cerebrovascular events and may not directly target the deficits caused by PPA. In addition, complete administration of these tests and the interpretation of their results require considerable time and skill.

The Clinical Dementia Rating (CDR) is another widely used scale for the cognitive-linguistic assessment of patients with various degenerative syndromes that determines the severity of their cognitive deficits and performance in activities of daily living. The language assessment included in this scale cannot provide a comprehensive picture of the language functioning of PPA patients. This means that although this instrument has been supplemented by language box with subtests for phrase length, word choice, word finding, reading and listening comprehension and grammar, it only provides a total score without any further details about the linguistic status of the assessed individual; making it useless for PPA individuals (Morris, 1993).

A comprehensive yet concise test that can be administered in clinical settings and is suitable for sampling core features of each PPA syndrome is clearly needed. However, designing PPA-specific tests has not been as prolific as it is in classic aphasia syndromes. As a result, aforementioned criterion-referenced aphasia batteries, i.e. WAB and BDEA -both of which primarily target patients with aphasia- have been used for the assessment of language performance in patients with PPA (Mesulam, 2003; Sapolsky et al., 2011). Therefore, a structured and semi-qualitative clinical instrument that is specifically designed for application in the population of PPA subjects can help clinicians rate and provide a more complete clinical picture of clients with PPA.

There are currently three available tests (Table 2) which have been specifically developed for PPA; Progressive Aphasia Severity Scale (PASS) (Sapolsky, Domoto-Reilly, & Dickerson, 2014), Progressive Aphasia Rating Scale (PARIS) (Saade et al., 2015), and Progressive Aphasia Language Scale (PALS) (Leyton et al., 2011).

**Table 2.** Summary features of Progressive Aphasia Language Scale (PALS), Progressive Aphasia Rating Scale (PARIS), and Progressive Aphasia Severity Scale (PASS)

**Test**	**Authors**	**Speech/Language Domains**	**Rating Range and Score**	**Assessment Method**	**Administration Time (Minute)**
PALS	Leyton et al. (2011)	1. Motor speech 2. Grammar 3. Naming 4. Single-word repetition 5. Single-word comprehension 6. Sentence repetition 7. Sentence comprehension	0: Absent 1: Subtle or questionable impairment 2: Mild but definitely present impairment 3: Moderate or severe impairment	Clinician’s Judgment about patient’s spontaneous speech, specific language tasks	NS^*^
PARIS	Saade et al. (2015)	1. Designation 2. Denomination 3. Single-word repetition 4. Sentence repetition 5. One minute categorical and lexical fluencies 6. Writing and reading of irregular words 7. Verb grammar 8. Facial-oral apraxia	Not mentioned; yields a total score of 55	Not mentioned	10
PASS	Sapolsky et al. (2014)	1. Articulation 2. Fluency 3. Syntax and grammar 4. Word retrieval and expression 5. Repetition 6. Auditory comprehension 7. Single word comprehension 8. Reading 9. Writing 10. Functional communication	0: Normal 0.5: Questionable or very mild impairment 1: Mild impairment 2: Moderate 3: Severe	Clinician’s Judgment about patient’s overall impairment based on structured interview and specific language tasks	NS^*^

* NS: Not Specified

PALS compared to PARIS and PASS is simpler and shorter and has better criterion validity which makes it a suitable bedside tool for detecting the different speech/language features of PPA variants.

The original version of PALS was developed and validated in English. Later, it was translated into Spanish (Gil-Navarro et al., 2013). However, to the best of our knowledge, no attempt has been made to develop a Persian version of PALS.

Persian-speaking speech-language pathologists and other clinicians working in the field of neurodegenerative disorders must be equipped with such instruments to specify PPA type and to reveal the signs and symptoms of the disorder in the initial phase. Due to the lack of standardized tests for assessing language characteristics of individuals with PPA in the clinical and research settings, the present study aimed to translate and culturally adapt the PALS into Persian language and assess the intra-rater reliability and discriminant validity of the Persian PALS in people with PPA.

## 2. Methods

### 2.1. Translation and cultural adaptation

Before applying PALS in clinical settings in Iran and Persian speaking countries, it must be translated into Persian and adapted culturally. To match the original and Persian version of the scale, following standard translation guidelines is necessary. Accordingly, the translation process of PALS was carried out following the guidelines proposed by the International Society for Quality of Life Assessment (Beaton, Bombardier, Guillemin, & Ferraz, 2000).

The translation process included the following phases: In phase 1, two Speech-Language Pathologists (SLP); T1 and T2, whose mother language was Persian and were fluent in English, translated the English version of PALS to Persian. Two expert linguists were also consulted.

At the completion of the first translation phase, translated scale by T1 was handed over to T2 and vice versa to score the difficulty level of translations according to a 100-point Likert-type scale, where 0 means easily understood, and 100 means highly difficult to understand. Each item scored >30 was considered as a difficult-to-understand translation and sent back to the initial translator for revision. Then, another two independent translators, fluent in English with no medical or clinical experience were asked to rate the translation quality in terms of clarity and simplicity. If items scored <90 on a 100-point Likert-type scale, the translated scale was sent back to the initial translator for reconsideration. No item was scored <90.

In phase 2, an expert panel including 3 translators, i.e. two speech-language pathologists and an experienced methodologist, reviewed the original English PALS and forward translations and produced the consensus version (T12). For phase 3, the consensus Persian version of PALS was back-translated to English by a bilingual translator. During phase 4, the expert panel reviewed all documents including original English, forward translations, consensus version of Persian PALS, back translation, and finally approved it for field testing. In phase 5, ten SLPs working in the field of dementia diagnosis and treatment were included for field testing trial. They were asked to rate the pre-final Persian PALS in terms of the clarity, fluency, comprehensibility, and the sociocultural suitability according to a 4-point scale, in which 0 represented the absence of an attribute and 4 indicated the presence of that feature. If any item was rated semantically or culturally unsuitable by 80% of the respondents, the discrepancies were discussed in the expert panel to apply revisions. In phase 6, the expert panel reviewed all documents and feedback from SLPs provided in the field testing trial and produced the final version of the Persian PALS (PALS-P).

### 2.2. Participants

Patients with the following criteria were included in the study: 1. PPA diagnosed by a neurologist, expert in the field of neurodegenerative disorders; 2. Fulfillment of the international consensus criteria proposed by Gorno-Tempini et al., (2011) assessed by an expert SLP; 3. Aged >50 years ; 4. Less than 3 years since the PPA onset; 5. Absence of severe psychological, neurological, and cognitive disorders. As an ethical rule, all participants gave written informed consent either themselves or through their next of kin, prior to study initiation. Ten Subjects with PPA and 20 neurologically healthy subjects were recruited from Yaadmaan Outpatient Clinic for Memory Disorders, Tehran, Iran.

### 2.3. Instrument

The outcome measure in this study was the examiner-based Persian version of PALS (PALS-P). The Progressive Aphasia Language Scale (PALS) consists of spontaneous speech and clinical tasks. The former is divided into motor speech disorder, agrammatism, and word retrieval deficits. The latter consists of naming, single-word repetition, single-word comprehension, sentence repetition, and sentence comprehension. In the revised version of the scale used in this study, the word retrieval subtest was removed and phonological error subtest was added (Leyton et al., 2011). The possible score for each subtest ranges from 0 (indicating no apparent disorder) to 3 (indicating severe disorder) (Leyton et al., 2011).

### 2.4. Procedure

Each patient was evaluated in a quiet room with minimal visual and auditory distractions by an experienced SLP familiar with the PALS. The instructions for each part of the test were completely explained to the participant. In the first part of the test, the patient was required to speak freely on topics such as travel and hobbies for 15 minutes. The patient’s speech was recorded to be later analyzed for any evidence of core features, including motor speech disorders, agrammatism and phonological errors. In the naming task, the patient was required to point to the line drawings of animals and household objects and name them one by one. The responses were transcribed in a response form. In the single-word repetition and comprehension tasks, the patients were asked to repeat increasingly more lengthy and complex words and to define some of the previously named words, respectively. In the sentence repetition task, several sentences of increasing length and syntactic complexity were presented to the patients to be repeated immediately as exactly as possible. To evaluate PALS-P intra-rater reliability, the test was re-administered two weeks later with the same patients. The same procedure was followed with the healthy subjects.

### 2.5. Statistical analysis

The normality of the data was tested by the Kolmogorov-Smirnov test. The Kruskal-Wallis test was used for discriminative validity analysis. The intraclass correlation coefficient (ICC agreement, 2-way random effect model) was used to determine the intra-rater reliability. A minimum of 0.7 was regarded acceptable for reliability (Terwee et al., 2007). All statistical tests were performed with SPSS v.17 (SPSS Inc., Chicago, IL, USA). Statistical significance was set at an alpha level of 0.05 for all analyses.

## 3. Results

In this study, 10 patients with PPA (4 males and 6 females; mean [SD] age=61.7(7.2) years, range=57–78 years) and 20 healthy subjects (10 males and 10 females; mean [SD] age=62.4(5.2) years, range =54–78 years) were selected. The mean (SD) duration between the onset of the PPA and participation in the study was 14.7 (5.4) months (range 9 to 24 months).

### 3.1. Discriminant validity

Statistical analysis of the data revealed significant differences between groups of subjects with PPA and healthy subjects in all subtests (P<0.001) (Table 3).

**Table 3.** Median (Interquartile range, IQR) for subtests of Persian Progressive Aphasia Language Scale (PALS-P) scores

**PALS-P Scale**	**Healthy Subjects, Median (IQR) (n=20)**	**Subjects With PPA, Median (IQR) (n=10)**	KWT^*^, P
Motor Speech Disorders (MSD)	0.00	2.50(1.25)	<0.001
Phonological errors	0.00	1.00(1.25)	<0.001
Agrammatism	0.00	0.00(1.00)	<0.001
Naming	0.00(1.00)	2.00(1.00)	<0.001
Single-word repetition	0.00	0.00(1.00)	<0.001
Single-word comprehension	0.00	1.00(1.25)	<0.001
Sentence repetition	0.00	1.00(0.7.5)	<0.001
Sentence comprehension	0.00	1.00(0.00)	<0.001

* KWT: Kruskal-Wallis Test

### 3.2. Intra-rater reliability

The intra-rater reliability coefficients for the all subtests of PALS-P (range=0.81–1.0, P<0.001) were acceptable (Table 4).

**Table 4.** ICC (95% CI) value for Intra-rater reliability of PALS-P sub-tests

**PALS-P Scale**	**ICC**	**95% CI**	**P**
Motor Speech Disorders (MSD)	0.85	0.52–0.96	<0.001
Phonological errors	0.88	0.52–0.96	<0.001
Agrammatism	1	1	<0.001
Naming	1	1	<0.001
Single-word repetition	0.85	0.52–0.96	<0.001
Single-word comprehension	0.88	0.60–0.97	<0.001
Sentence repetition	1	1	<0.001
Sentence comprehension	0.81	0.11–0.85	=0.048

## 4. Discussion

The present study aimed to develop the Persian PALS and validate PALS-P in a sample of Persian-speaking people with PPA. This pilot study found that the PALS-P has satisfactory intra-rater reliability and validity.

### 4.1. Translation and adaptation

There was no major problem with the translation and cultural adaptation of the English PALS into Persian. The standard methodology followed in the translation process ensured the content equivalence of the PALS-P with the original English version (Leyton et al., 2011).

Considering the fact that PALS is a language scale designed for the assessment of core linguistic features represented by patients with PPA, the translation of its verbal stimuli, especially the items chosen for single-word repetition, sentence repetition, single-word comprehension, and naming tasks, required meticulous attention.

In single-word repetition subtest, through which the length and complexity of stimulus words and phrases gradually increase, literal translation was avoided and suitable Persian equals were introduced into the translation. For example, ‘Chrysanthemum’ is a phonologically complex word in English selected as an item of single-word repetition task, but its equivalent word in Persian is much simpler (/davudɪ/). Thus, a totally different word was selected to meet the phonological complexity demand in this task (/doʃ′mæntæ′rɒ:ʃi:/). These stimuli consistent with the original instrument were used in the single-word comprehension task.

For translating sentence repetition task items, the original sentences were first analyzed by two linguists to determine their main phonological and syntactic properties and appropriate sentences with equal properties were chosen from Persian language. For example, the Persian equivalent for the sentence ‘Six small boys built a sizeable snowman’ is ‘/′tɒ:ʤer ′to ′ʧe te′ʤɒ:ræt ′mi:koni/’ (Businessman, what do you trade?).

Some minor modifications were made in the scoring system of the test. For example, the word ‘church’ was used as an example of the consonant /ʧ/in the definition of grouping. However, the Persian word for church (i.e. / kelɪsa/) is devoid of affricatives. So the Persian word for hammer (i.e. /′ʧækkoʃ/) replaced it.

The naming subtest stimuli were first translated literally from English into Persian and then their frequency and familiarity were evaluated. Since the translated words had the same frequency and familiarity rates as their English counterparts, no further changes were made (Appendix 1).

The translation process verified the face and content validity of the PALS-P for use in new setting with Persian language and culture (Beaton et al., 2000).

### 4.2. Discriminant validity

As expected, patient scores were worse than those of healthy subjects in all subtests of PALS-P. This indicates that the Persian version of PALS is able to discriminate between patients with PPA and healthy individuals.

### 4.3. Intra-rater reliability

The ICC for all subtests of PALS-P was excellent. This indicates that the test results have acceptable consistency and the mean score of each subtest does not have a significant difference in the two administrations of PALS-P. This shows that repeated administrations of PALS-P in patients with PPA yielded similar and relatively identical results. The sentence comprehension subtest seems to be borderline significant that may be explained by the fact that this subtest depends primarily on working memory abilities more than other subtests. Patients with non-fluent variant have a true syntactic deficit, which has been demonstrated using task that do not put high demand on working memory load (Weintraub et al., 2009). The intra-rater reliability has not been reported for the original version of PALS-P (Leyton et al., 2011).

Finally, the adaptation and validation of PALS-P according to the previously described standard methodology made it a suitable tool for the assessment of Persian-speaking people with PPA. The results of this preliminary study support the psychometric properties of face, content, and discriminative validity as well as the intra-rater reliability of PALS-P.

Despite the fact that the study fulfilled its objectives, there were some unavoidable limitations. First, the sample size of patients was small (due to slow recruitment and limited time to perform the study). Second, it would be ideal to include all three types of PPA in the validation process of PALS-P and other psychometric characteristics such as diagnostic accuracy of sensitivity and specificity, construct validity, criterion validity be determined in future investigations. Future studies should include larger sample size aiming at inter-rater reliability, and validating the PALS-P across PPA types.

## Appendix 1. This section provides information on the pronunciation of Farsi names for each item based on International Phonetic Alphabet (IPA), English translations of the names of the items in naming, comprehension and repetition sub-test. It is a supplementary Table and should only be available online.

**Table:**

**Subtest**	**Original Item**	**Farsi Names (IPA)**	**Translation in English**
Word repetition	‘Banana’	/′ʔɒnɒnɒs/	Pineapple
	‘Potato’	/tu′ti:′hɒ/	Parrots
	‘Methodist’	/sa′ʔɒdæt/	Prosperity
	‘Artillery’	/′ɡɒzɡerefte′ɡi:/	Biting
	‘Perimeter’	/sær′bɒz′xɒne/	Barracks
	‘Caterpillar’	/dust′jɒbi:/	Finding friends
	‘Catastrophe’	/for′sæt′su:zi:/	Wasting opportunity
	‘Chrysanthemum’	/doʃ′mæntæ′rɒ:ʃi:/	Making enemies
Sentence repetition	‘No ifs ands or buts’	/be′dune ′hi:ʧ æmmɒ ′væ ′ʔæɡæri:/	Without any buts and ifs
	‘The Chinese fan contained a rare emerald’	/′ʔæz ′særʔejn tɒ: xoj ′rɒ:nændeɡi: ′kærdæm/	I drove from Sar-eyn to Khoy.
	‘Six small boys built A sizeable snowman’	/′tɒ:ʤer ′to ′ʧete′ʤɒ:ræt ′mi:koni/	Business man, what do you trade?
	‘The lady delivered some delicious gingerbread’	/′du:ste′dɒ:neʃ′mændæm dæ′vɒ:zdæh me′dɒ:d ′be ′mæn ′dɒ:d/	My scientist friend gave me twelve pencils.

## References

[B1] Beaton D. E. Bombardier C. Guillemin F. Ferraz M. B. (2000). Guidelines for the process of cross-cultural adaptation of self-report measures. Spine, 25(24), 3186–91. doi: 10.1097/00007632-200012150-00014 11124735

[B2] Gil-Navarro S. Lladó A. Rami L. Castellví M. Bosch B. Bargalló N. (2013). Neuroimaging and biochemical markers in the three variants of primary progressive aphasia. Dementia and Geriatric Cognitive Disorders, 35(1–2), 106–17. doi: 10.1159/000346289 23392204

[B3] Goodglass H. Kaplan E. Barresi B. (2000). Boston diagnostic aphasia examination-(BDAE-3). San Antonio: Psychological Corporation.

[B4] Gorno Tempini M. L. Hillis A. E. Weintraub S. Kertesz A. Mendez M. Cappa S. F. (2011). Classification of primary progressive aphasia and its variants. Neurology, 76(11), 1006–14. doi: 10.1212/wnl.0b013e31821103e6 21325651 PMC3059138

[B5] Kertesz A. (1982). Western aphasia battery test manual. San Antonio: Psychological Corporation.

[B6] Leyton C. E. Hodges J. R. (2014). Differential diagnosis of primary progressive aphasia variants using the international criteria. Aphasiology, 28(8–9), 909–21. doi: 10.1080/02687038.2013.869306

[B7] Leyton C. E. Villemagne V. L. Savage S. Pike K. E. Ballard K. J. Piguet O. (2011). Subtypes of progressive aphasia: Application of the international consensus criteria and validation using β-amyloid imaging. Brain, 134(10), 3030–43. doi: 10.1093/brain/awr216 21908392

[B8] Mesulam M. M. (1982). Slowly progressive aphasia without generalized dementia. Annals of Neurology, 11(6), 592–8. doi: 10.1002/ana.410110607 7114808

[B9] Mesulam M.-M. (2003). Primary progressive aphasia: A language-based dementia. New England Journal of Medicine, 349(16), 1535–42. doi: 10.1056/nejmra022435 14561797

[B10] Mesulam M. M. Dickerson B. C. Sherman J. C. Hochberg D. Gonzalez R. G. Johnson K. A. (2017). Case 1-2017. New England Journal of Medicine, 376(2), 158–67. doi: 10.1056/nejmcpc1613459 28076711 PMC5264551

[B11] Mesulam M. M. Wieneke C. Thompson C. Rogalski E. Weintraub S. (2012). Quantitative classification of primary progressive aphasia at early and mild impairment stages. Brain, 135(5), 1537–53. doi: 10.1093/brain/aws080 22525158 PMC3577099

[B12] Morris J. C. (1993). The Clinical Dementia Rating (CDR): Current version and scoring rules. Neurology, 43(11), 2412. doi: 10.1212/wnl.43.11.2412-a 8232972

[B13] Saade Y. M. Ferrieux S. Arbizu C. Saratxaga A. A. Hermann B. Henry Amar F. (2015). Paris: A new comprehensive language tool for the diagnosis and categorization of primary progressive aphasia. Alzheimer’s & Dementia, 11(7), P130–1. doi: 10.1016/j.jalz.2015.07.042

[B14] Sapolsky D. Domoto Reilly K. Dickerson B. C. (2014). Use of the Progressive Aphasia Severity Scale (PASS) in monitoring speech and language status in PPA. Aphasiology, 28(8–9), 993–1003. doi: 10.1080/02687038.2014.931563 25419031 PMC4235969

[B15] Sapolsky D. Domoto Reilly K. Negreira A. Brickhouse M. McGinnis S. Dickerson B. C. (2011). Monitoring progression of primary progressive aphasia: Current approaches and future directions. Neurodegenerative Disease Management, 1(1), 43–55. doi: 10.2217/nmt.11.2

[B16] Terwee C. B. Bot S. D. M. de Boer M. R. van der Windt D. A. W. M. Knol D. L. Dekker J. (2007). Quality criteria were proposed for measurement properties of health status questionnaires. Journal of Clinical Epidemiology, 60(1), 34–42. doi: 10.1016/j.jclinepi.2006.03.012 17161752

[B17] Weintraub S. Mesulam M.-M. Wieneke C. Rademaker A. Rogalski E. J. Thompson C. K. (2009). The northwestern anagram test: Measuring sentence production in primary progressive aphasia. American Journal of Alzheimer’s Disease & Other Dementiasr, 24(5), 408–16. doi: 10.1177/1533317509343104 PMC2836907 19700669

